# The Thermal Conductivity of 3D Printed Plastic Insulation Materials—The Effect of Optimizing the Regular Structure of Closures

**DOI:** 10.3390/ma13194400

**Published:** 2020-10-02

**Authors:** Beata Grabowska, Jacek Kasperski

**Affiliations:** Faculty of Mechanical and Power Engineering, Wroclaw University of Science and Technology, Wybrzeze Wyspianskiego 27, 50-370 Wroclaw, Poland; jacek.kasperski@pwr.edu.pl

**Keywords:** heat transfer, thermal insulation, 3D printing, closure, structure

## Abstract

In the interest of environmental protection, attention should be paid to improving energy efficiency, through the use of appropriate insulations. They can be used in the construction industry, for plastic window frames, and the thermal insulation of buildings. It is also possible to use these materials in the electronics industry, for hermetic casings of devices, in the aviation industry, as well as in the food industry, as collective packaging for frozen food. The technology of using additive 3D printing to create prototype insulating materials made of plastic is proposed in this article. Multi-layer materials, with quadrangle, hexagonal, and triangle closures were designed and printed. A mathematical model was developed, and then experimentally verified. Quadrangle and hexagonal structures were shown to be useful, and triangle structures to be of little use. The optimal size of closure was determined to be 10 mm, with no convection, and 6 mm, with possible convection. The lowest thermal conductivity of the insulation was 0.0591 W/(m·K) for 10 mm single-layer quadrangle and hexagonal closures with an insulation density of 180 kg/m^3^.

## 1. Introduction

Fossil fuels are the main source of energy, and from year to year they increase environmental pollution, causing global warming, etc. It therefore seems necessary to find new strategies, which include, among others, renewable energy sources, or the improvement of energy efficiency. Over 60% of the total energy produced is consumed by industrial and residential buildings [1,2]. The many possibilities of reducing energy losses in the construction sector include the optimization of a building’s design by improving, among other things, its external insulation, or the structure of window frames [3]. The thermal properties of windows are very important for energy-efficient buildings. Windows, in general, account for around 30–50% of transmission losses through the building, even if the area fraction of the coating is much smaller. While window frames typically account for 20–30% of the total window surface, their impact on the overall heat transfer coefficient of the window can be much greater. This effect is even more significant in windows with low conductivity (highly insulating), which contain glazing with a very low conductivity [4].

3D printing, i.e., additive manufacturing (AM), is the process of creating real and three-dimensional objects using virtual model designs. The process is additive, and involves forming successive layers of material in the desired way. AM allows the production of non-standard parts from metals, ceramics, and polymers without the need for molds or the processing that is typical for conventional production, when using traditional loss technologies [5,6]. While conventional manufacturing is subjected to the processing constraints that are associated with industrial mass production, AM enables a faster conversion from the design stage, and production of custom objects that are tailored to the requirements of individual people and specific applications [6,7,8]. This flexibility in creation, and the incredible decrease in the cost of 3D printing, has led scientists from around the world to become interested in this technology as a tool. Among the various technologies included in AM, the most commonly used are selective laser sintering (SLS), selective laser melting (SLM), and fused deposition modeling (FDM) [9].

At the beginning of the 70s, in the twentieth century, 3D printing was a young, and not widespread technology, and therefore only reserved for a few. Over the past few decades, the situation has changed dynamically, which has resulted in the fact that 3D printing has become widely available. The application of 3D printing technology is also increasing in medicine [5,10,11,12]. Additive manufacturing has enabled the production of patient-specific medical implants, with gradations in their internal geometry, which is otherwise difficult and too expensive to manufacture. This is due to the possibility of accurately mapping the desired elements. 3D printing allows human organs to be accurately replicated, which helps in the education and training of doctors. Thanks to medical imaging, three-dimensional models of parts of a patient’s body can also be created, which can then help in planning surgeries. There are also known cases of printing endoprostheses for individual patients, which replace or support the functions of organs. The most common are hip and knee implants, hearing aids, or skin, bone, and tissue prostheses. Such a solution is often cheaper when compared to traditional products [5,13]. The AM technique facilitates the fabrication of any geometrical or complex-shaped structures that would not be easily produced when using conventional fabrication methods without additives. Therefore, 3D printing is a potential salvation, with regards to the medical materials that are missing at certain times [14].

Other sectors that use the potential of 3D printing are the aviation, marine, automotive, and construction industries. Special materials such as sandwich composites, which are widely used in these industries, are taking on a new meaning thanks to 3D printing technology. Composites with the so-called sandwich structure [7] deserve special attention. Layered structures have rigid outer layers and a low-density core in their structure. The core can be made of alternative materials, but is usually in the form of closed, air-like material closures, e.g., foam or periodic structures [7,15]. The most commonly used layered materials can be divided into four types: cellular foam, honeycomb, corrugated cardboard, and balsa [7]. Honeycomb structures consist of plates or sheets that form the elementary edges of a closure. They can be arranged to form triangle, square, hexagonal, or other related shapes. Their unit closures are repeated in two dimensions in order to form a solid body. All honeycombs are closed cellular structures [16].

In the literature [17,18,19], much attention has been paid to the use of sandwich panels with a honeycomb core. Such cellular cores are rigid, light, and absorb high energy under the influence of impacts and shock waves, which is particularly important when used in sports equipment, as well as in the automotive and aviation industries [20,21,22]. According to [5], however, they have some special properties due to their closed-cellular architecture, i.e., they not only retain gas, which leads to low thermal conductivity, but also retain moisture. Moisture trapped in closed-cellular cores increases their weight and shifts the center of gravity, and this can be solved using open-cellular cores [23].

Light sandwich panels are now widely used due to their high bending stiffness to weight ratio, excellent thermal insulation, and high energy absorption capacity [7,23]. The core architecture is complex, and has intricately shaped ligaments and density gradients [23]. This is accompanied by the progress in advanced manufacturing technologies, such as additive production (3D printing) and laser cutting [24]. In the literature [25,26,27,28], authors have used additive manufacturing to create layered structures with architectural cores. They showed, among other things, that the properties of cellular materials are not only determined by solid components, but also by the spatial configuration of voids and solids, i.e., the cellular architecture. The change in the cellular architecture provides unlimited ways of achieving the desired material properties. This brings a number of advantages when compared to conventional sandwich structures, with hexagonal, honeycomb, or foam cores. Simultaneously, computing power and computational methods have been sufficiently improved in order to enable the design of materials and structures with complex cellular architecture, and which are optimized for specific applications [23].

## 2. Materials and Methods

### 2.1. Practical Use Multilayer Insulation Material Made of Plastic with Thin Walls

**Figure 1:**
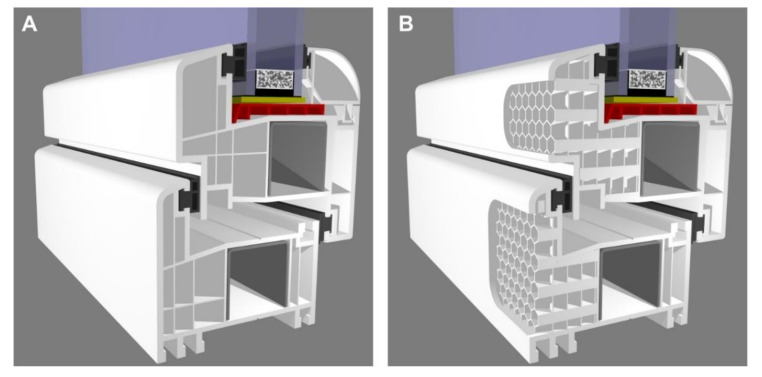
Cross-section of a plastic window frame: (**A**) typical, (**B**) external parts filled with the 3D printed exemplary structure considered by the authors.

Over the past few years, the authors have conducted research on obtaining an effective construction of insulating material. Multilayer composites were made of thin sheets of polypropylene foil, welded together in order to form rectangular oblong air cells [15,29,30]. Literature studies prompted the authors to produce composite materials using 3D printing technology. The availability, simplicity, as well as satisfactory precision of 3D printing technology allow it to be successfully used as a tool when conducting engineering experiments. There is a wide range of possible applications of 3D printed insulation structures. These structures can be filled with air or other gases, e.g., argon, and there can also be a vacuum inside such a structure (the so-called vacuum insulated panel (VIP) materials). Structures filled with air could be used in the construction industry for plastic window frames (see Figure 1) and the thermal insulation of buildings. It is also possible to use these materials in the electronics industry for the hermetic casings of devices, in the aviation industry, as well as in the food industry as collective packaging for frozen food.

The possibility of printing almost any shape of object allows the influence of the shape of the structure on its properties to be determined. Therefore, an important issue for the authors when designing a new structure of insulating material was, first of all, to appropriately select the geometry of the plastic walls that form the internal regular closure.

### 2.2. Design And 3D Printing of Multilayer Insulation

The authors assumed that the comparison should be based on a four-variant set of sample structure module sizes: D = 4, 6, 8, 10 mm; and a three-variant set of the number of structure layers: n = 1, 2, 3. The spatial modeling program (3D CAD) Autodesk Inventor was used to design the virtual geometry of the object as a closure that is closed by wall surfaces.

**Figure 2:**
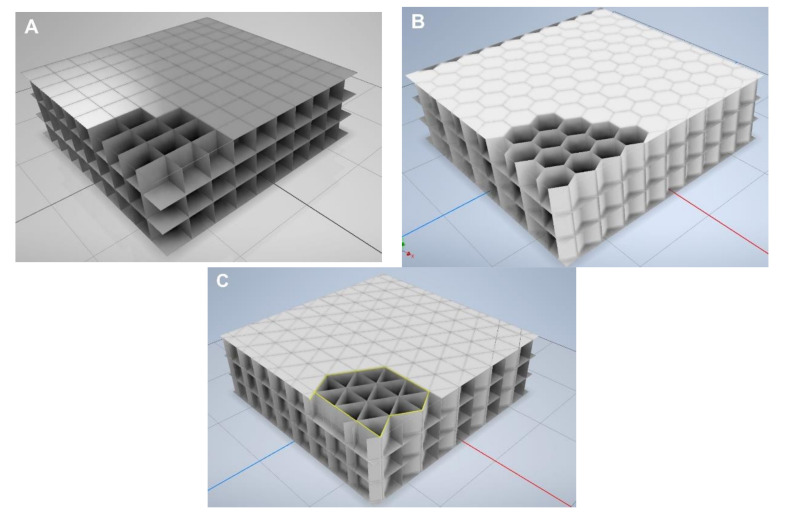
Design of air partitions with different regular closures. (**A**) square shape, (**B**) hexagonal shape, (**C**) triangular shape.

Each of the designed models was a cuboid with periodically arranged regular closures. Due to the types of designed closures, air partitions were divided into three groups, for three-layered samples, as can be seen in Figure 2.

The designed air partitions had regular closures that were arranged in a rectangular pattern, and had a square cross-section for the first group, a hexagonal cross-section for the second group, and an equilateral triangular cross-section for the third group. An SLS 3D printer was used to print the designed models of the prototype insulation material. The current SLS printing technology does not allow a multi-layer insulation, the idea of which is presented in Figure 2, to be printed. This is due to the fact that it is not possible to remove the powder from the space enclosed by walls. For this reason, the authors printed single layers with a thin base, from which it was easy to remove the powder. Afterwards, the individual layers were glued together to form a multi-layer insulation, and a thin closing cover was added on top.

**Figure 3:**
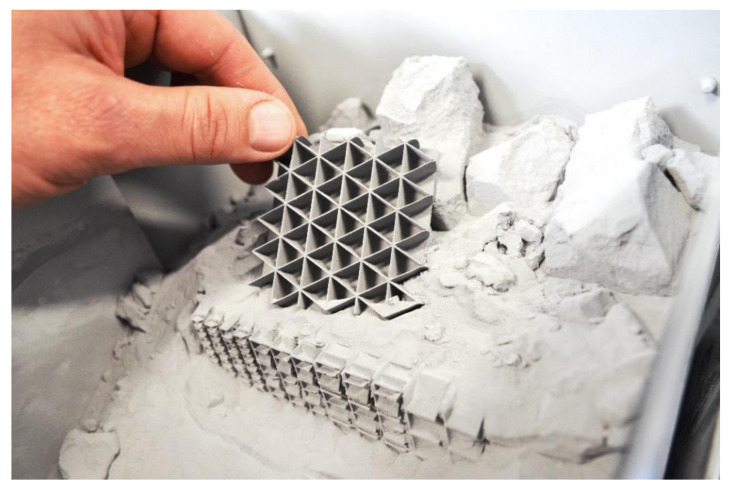
A single layer of a printed structure when being taken out of the 3D printer.

Selective laser sintering (SLS) is a universal technique that uses a laser beam to sinter powdered materials and create three-dimensional objects. Thanks to its high printing precision, this method is suitable for making molds or prototypes that must match their patterns. PA-12 (polyamide) powder was used as the printing material. Several factors influenced the final appearance of the designed models of the prototype insulation material. The first that should be mentioned was the necessity to reduce the mass fraction of walls made of PA12 plastic. Another factor that was taken into account was the physical limitations of the printer, and its parameters regarding the precision of printing, which determined the minimum thickness of the walls made of plastic. The design of the air partitions assumed a wall thickness of 0.075 mm, but after printing their thickness was about 0.5 mm. The 3D printed thermal insulation material is presented in Figure 3.

The next part of the article presents a mathematical model of the occurring heat transfer processes, which were compared with the results of the experimental studies.

### 2.3. Mathematical Model

Heat transfer in insulation is a complicated issue. This is because thermal insulations usually consist of many components, and their structure is complex. The basic quantity that is used to assess the quality of insulation is its thermal conductivity [1], and additionally its density.

In larger air-filled closures, thermal conductivity increases. This is caused by the movement of air, which is a result of the heating of the air on the warmer bottom surface and its cooling on the colder top surface (convection).

**Figure 4:**
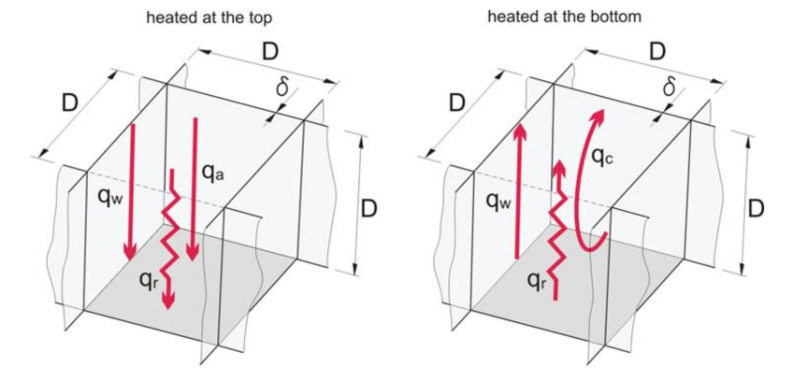
Diagram of the processes of heat exchange that occur in the air partition, using the example of the quadrangle structure [29,30].

As already proved in the previous considerations [15,29,30], the heat flux in a regular structure is calculated as the sum of (Figure 4):

q_w_—Heat conduction along plastic walls, W/m^2^,
q_a_—Heat transfer through air,
q_c_—Convective heat transfer, depending on the Rayleigh number value, W/m^2^,
q_r_—radiation between layers, W/m^2^.

The total thermal resistance for the top heating variant (no convection) is calculated using the formula:

(1) $$R_{HT}=\frac{T_{H}-T_{C}}{q_{w}+q_{c}+q_{r}}$$

where:

*T_H_*—Temperature of hot side, K
*T_C_*—Temperature of cold side, K

The total thermal resistance for the bottom heating variant (convection may occur) is calculated using the formula:

(2) $$R_{HB}={\frac{T_{H}-T_{C}}{q_{w}+q_{c}+q}}_{r}$$

The total thermal conductivity for insulating material consisting of n layers is equal to:

(3) $$K_{i}=\frac{n\cdot D}{R}$$

where:

*n*—Number of layers,
*D*—Dimension of structure closure, m
*R*—Thermal resistance, m^2^·K/W

**Figure 5:**
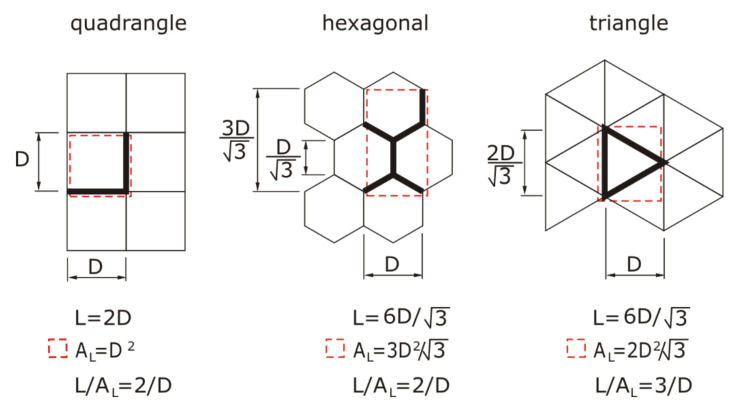
The dependence between the length of the walls and the type of structure in relation to its base area. The red dashed line means the heat transfer area, with walls that have length, L.

The heat conduction along the walls of the insulation is described by formula:

(4) $$q_{w}=\frac{L\cdot \delta }{A_{L}\cdot D}\cdot K_{w}\left(T_{H}-T_{C}\right)$$

where:

*L*—Wall length, m
*A_L_*—Heat transfer area with walls that have length, L, m^2^
*K_w_*—Thermal conductivity of the wall material, W/(m·K)

where, depending on the type of structure (Figure 5), the *L/A_L_* value (wall length per unit area of insulation) is equal to:

*L/A_L_* = 2/D for the quadrangle and hexagonal structures,
*L/A_L_* = 3/D for the triangle structure.

Since the manufacturer of the powder for SLS technology does not provide its exact thermal conductivity, it was measured experimentally using a Poensgen apparatus, and described in the “Experiments” section of the article. The printed flat block of material, with a thickness of 15 mm, had K_w_ = 0.023 W/(m·K), and this value was adopted for further calculations.

The conduction inside a closed air closure that was heated at the top was calculated from the formula:

(5) $$q_{a}=\frac{K_{a}}{D}\cdot \left(T_{H}-T_{C}\right)$$

where:

*K_a_*—Thermal conductivity of air, W/(m·K)

In the insulation that was heated at the bottom, instead of heat conduction, convection will occur, and then:

(6) $$q_{c}=\frac{Nu\cdot K_{a}}{D}\left(T_{H}-T_{C}\right)$$

where Nu depends on the Rayleigh number, according to the formula presented by Roshenow [31]. The equation describes convection in rectangular, parallel-sided closures, and was successfully applied by the authors in previous investigations [15,29,30]:

(7) $$Nu=1+{\left[1-\frac{1708}{Ra}\right]}^{*}\left[k_{1}+2{\left(\frac{Ra^{1/3}}{k_{2}}\right)}^{1-\ln\left(\frac{Ra^{1/3}}{k_{2}}\right)}\right]+{\left[{\left(\frac{Ra}{5803}\right)}^{1/3}-1\right]}^{*}$$

*—brackets indexes are taken into account in the equation when the parenthesis is positive. If however, it is negative zero is inserted in their place

where:

(8) $$k_{1}=\frac{1.44}{1+0.018/\mathrm{Pr}+0.00136/{\mathrm{Pr}}^{2}}$$

(9) $$k_{2}=75\cdot \exp\left(1.5\cdot {\mathrm{Pr}}^{-1/2}\right)$$

where:

(10) $$Ra=\frac{g\cdot \beta }{\nu \cdot \alpha }\cdot \left(T_{H}-T_{C}\right)\cdot D^{3}$$

The calculated values of Nu varied between 1 (lack of convection) and 2 (convection), whereas the Re number varied from 580 up to 6000, respectively.

For the analyzed insulation, the radiation heat flux *q_r_* between opposite parallel planes (heated and cooled) is equal to:

(11) $$q_{r}=\sigma \cdot \varepsilon \cdot \tau \cdot \left(T_{H}^{4}-T_{C}^{4}\right)$$

Radiative heat transfer in rectangular, parallel-sided closures includes heat transfer between cold and hot horizontal walls, as well as between horizontal and lateral vertical walls. The first of the described variants is easy to calculate, and was described by Equation (11), whereas the second variant is very difficult to calculate because it depends on the temperature of the vertical wall, which changes along its height. For this reason, it was decided to omit this computational issue.

The volume of the flat base and the lengths of the walls of the printed structures, presented earlier in Figure 5, were used to calculate the insulation density. Subsequent measurements of the mass of the printed insulation samples showed, however, that the samples were about 25% heavier than those in the theoretical calculations. For this reason, a permanent 25% (excess) weight correction was introduced into the computational model concerning the amount of plastic.

### 2.4. Experiments

In order to confirm the theoretical considerations, the authors experimentally determined the value of the thermal resistance for each of the previously described variants of the prototype insulation material.

**Figure 6:**
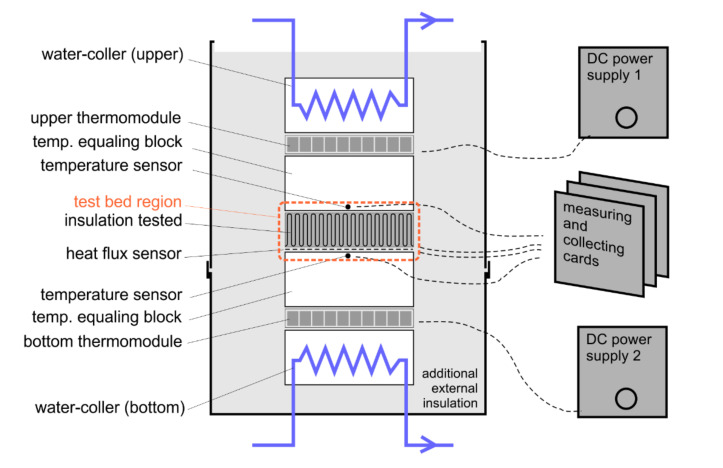
Schematic diagram of the thermoelectric version of the hot plate apparatus applied for experimental measurements.

**Figure 7:**
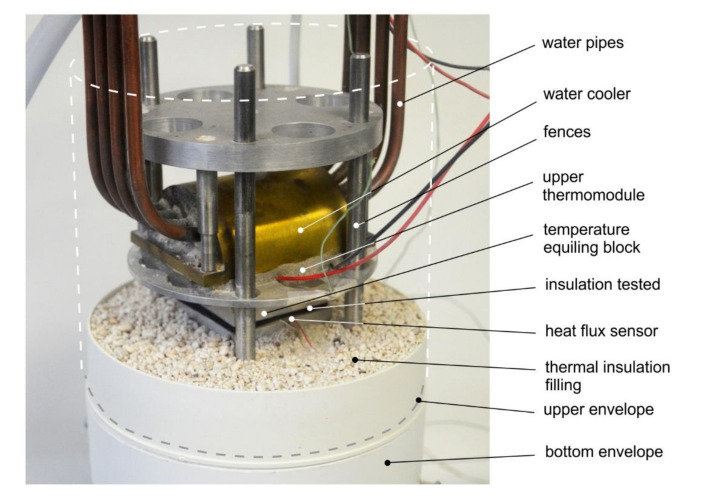
Photo of hot plate apparatus used by the authors (the upper envelope and insulation filling are removed for better visibility).

The thermal resistance measurements were carried out using the thermoelectric version of the Poensgen (sometimes named hot and cold plate) apparatus, which was built at the Wrocław University of Science and Technology [13,28,29]. The device contains two plates, in order to create a steady-state heat transfer. Its schematic diagram is presented in Figure 6 and its photo is presented in Figure 7. Two thermoelectric modules (upper and bottom) are cooled by water heat exchangers. Each thermomodule is supplied by a DC power station with regulated current. According to its polarization, the thermomodule can be switched on to work in heating or cooling mode.

The thermal power of the thermomodule changes according to the value of the electric current of its supply, and maintaining a constant value of the current enables a constant temperature in the vicinity of the test bed to be set. To equal the spatial temperature distribution of each side of the bed, 20 mm thick blocks of aluminum were used. For the temperature measurement, two K-type thermocouples were applied, and were located close to the test bed. A heat flux sensor (Omega HFS-4), mounted on one of the aluminum block sides of the tested insulation, was used to measure the heat flowing through it. Therefore, the test bed part of the apparatus enabled a temperature in the range from −30 to +50 °C (fluctuation of less than ±0.1 °C) to be reached. Uncertainty of thermal resistance for the measurements was estimated to be less than 8%.

Two modes of heat conduction through the prototype insulation material were taken into account in the experimental measurements:

(a) heated at the top mode,
(b) heated at the bottom mode.

For variant (a), the apparatus was switched to the mode with upper heating, and cooling using the lower thermomodule, and for variant (b) it was switched to the mode with lower heating, and cooling using the upper thermomodule.

Each measurement required thermal equilibrium to be established, and usually lasted about 45 min. Based on the measured values, the value of the thermal resistance of the printed insulation was calculated. The temperature of the heated and cooled sides of the device was kept at +20 °C for the hot side, and −20 °C for the cold side (fluctuation <0.1 °C). Both of the adopted values correspond to the typical working conditions of thermal insulation in the construction and food industries, as well as those used in the transport of frozen food.

## 3. Results

**Figure 8:**
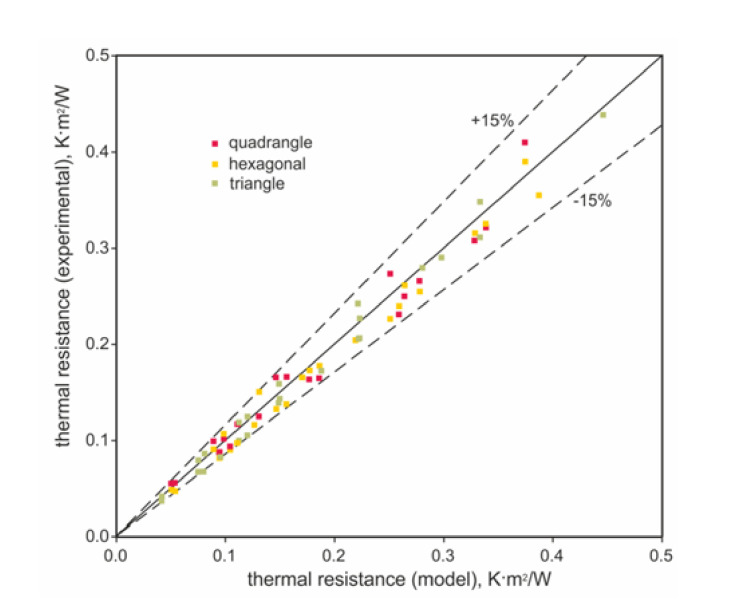
Comparison of the thermal resistance of insulation that was obtained from the calculations and experimental measurements.

All the calculations based on the set of Equations (1)–(11), as well as the experimental results, are presented in Table 1, and also in graphical version in Figure 8. It can be seen that the results of the experimental tests differ by no more than ±15% from the calculation results, while for the heated at the top mode (without convection) these differences are smaller, and do not exceed ±13%. It was found that the applied calculation model can be considered to be correct. It should be noted that the proposed heat transfer model concerns a relatively small share of radiative heat transfer. In the case of higher temperatures, the adopted model may not achieve the described accuracy. The authors did not perform experimental tests for higher temperatures of the tested thermal insulation.

Due to having a computational model, it was decided to perform calculations for the size of closures: D equal to 4, 5, 6, 8, and 10 mm; and for the number n equal to 1, 2, 5, and 10 layers. The obtained results of calculating the thermal conductivity were compared with the calculated material density, which is the subject of basic analyses for most thermal insulation materials. The best insulation materials are those with low thermal conductivity and low density.

**Figure 9:**
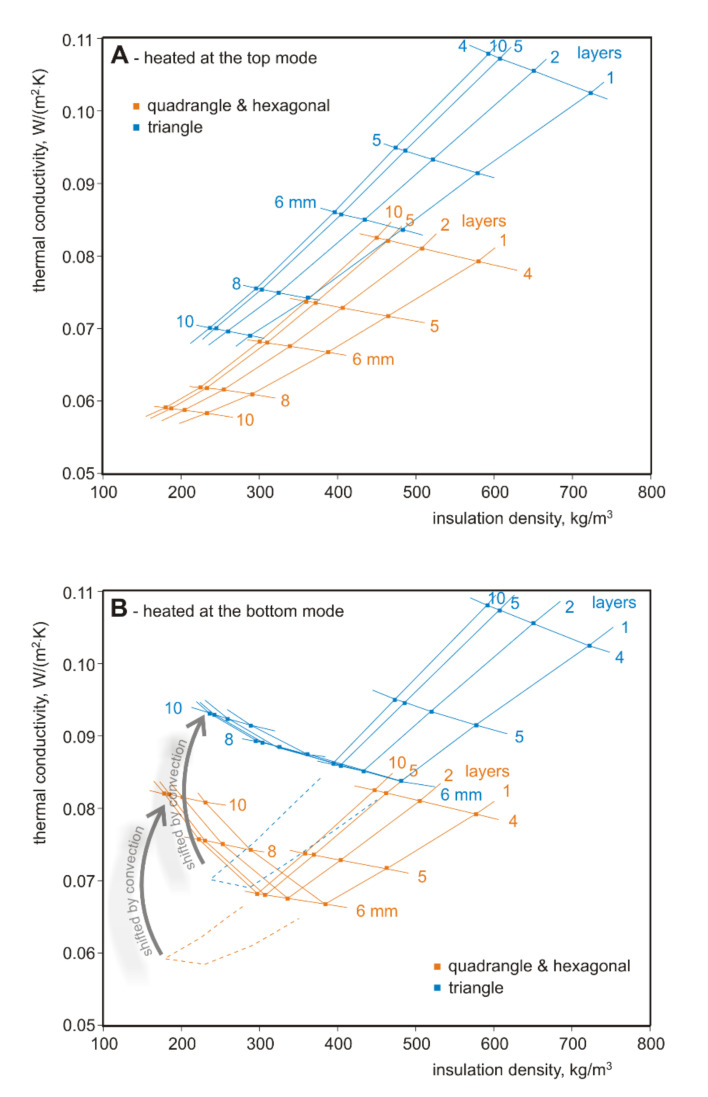
Thermal conductivity in relation to the density of the considered additively manufactured insulation. (**A**) heated at the top mode, (**B**) heated at the bottom mode.

The summary of the analysis results is presented in Table 2 and in Figure 9. Figure 9A shows the results for heating at the top mode, Figure 9B shows the results for heating at the bottom mode.

## 4. Discussion

It can be seen that for heating at the top mode, the lowest thermal conductivities and densities are for the quadrangle and hexagonal closures, and worse are the triangle closures. The larger the size of the closures, the lower the thermal conductivity and density. With more layers, the thermal conductivity increases slightly, but the density decreases. The lowest possible thermal conductivity is equal to 0.0591 W/(m·K), and occurs for single-layer quadrangle and hexagonal closures with a size of 10 mm at a density of 180 kg/m^3^. The lowest possible thermal conductivity for 10 mm single-layer triangle closures is equal to 0.0702 W/(m·K) at a density of 238 kg/m^3^.

It is visible that for for heating at the bottom mode and closure sizes above 6 mm there is a rapid increase in convection, which causes the thermal conductivity to increase (and not decrease as was the case with heating at the top mode, marked with dotted lines). As shown in Figure 9B, the left side of the graph visibly rises upwards, due to the increasing convection forces. The smallest thermal conductivities and densities are still found for the quadrangle and hexagonal closures, and are greater for the triangle closures. The lowest possible thermal conductivity is equal to 0.0682 W/(m·K) and occurs for 6 mm quadrangle and hexagonal single-layer closures at a density of 300 kg/m^3^. The lowest possible thermal conductivity is seen for 6 mm single-layer triangle closures and is equal to 0.0862 W/(m·K) at a density of 397 kg/m^3^.

## 5. Conclusions

The applied mathematical model of heat transfer was consistent with the results of the experimental tests, and the differences did not exceed ±15% (for temperatures +20 °C for the hot side, and −20 °C for the cold side). However, for heated at the top mode (without convection) the differences were smaller, and did not exceed ±13%. It was found that the applied computational model can be considered to be correct.

The lowest thermal conductivities could be obtained for he quadrangle and hexagonal closures, with the triangle closures obtaining worse conductivities. The larger the size of the closures, the lower the thermal conductivity and density.

For heating at the bottom mode, the presence of convection for closures with sizes above 6 mm was visible, and therefore they should not exceed this size.

In heating at the top mode, the lowest possible thermal conductivity of the insulation was equal to 0.0591 W/(m·K), and was available for single-layer quadrangle and hexagonal closures of 10 mm with an insulation density of 180 kg/m^3^.

In heating at the bottom mode, the lowest possible thermal conductivity of the insulation was equal to 0.0682 W/(m·K), and occurred for 6 mm quadrangle and hexagonal single-layer closures with an insulation density of 300 kg/m^3^.

For other researchers, the authors can recommend using quadrangle and hexagonal closures with dimensions smaller then 6 mm.

## Figures and Tables

**Table 1:** Comparison of calculation and experimental results of the thermal resistance of the insulation.

	Structure		Quadrangle				Hexagonal				Triangle			
		Number of layers	Dimension of closure, mm				Dimension of closure, mm				Dimension of closure, mm			
			4	6	8	10	4	6	8	10	4	6	8	10
			Thermal resistance, m^2^K/W				Thermal resistance, m^2^K/W				Thermal resistance, m^2^K/W			
Heated at the top	Model	1	0.050	0.090	0.131	0.171	0.050	0.090	0.131	0.171	0.039	0.072	0.108	0.144
		2	0.099	0.178	0.260	0.340	0.099	0.178	0.260	0.340	0.076	0.141	0.213	0.287
		3	0.147	0.265	0.389	0.509	0.147	0.265	0.389	0.509	0.113	0.210	0.318	0.429
	Experimental	1	0.055	0.099	0.125	0.165	0.048	0.091	0.151	0.166	0.042	0.079	0.118	0.143
		2	0.102	0.163	0.230	0.320	0.106	0.172	0.238	0.324	0.086	0.158	0.226	0.289
		3	0.166	0.248	0.354	0.470	0.132	0.260	0.354	0.476	0.124	0.242	0.310	0.437
	Discrepancies *, %	1	−8	−9	5	4	5	−1	−13	3	−7	−10	−9	1
		2	−3	9	13	6	−7	3	9	5	−12	−11	−6	−1
		3	−12	7	10	8	11	2	10	7	−9	−13	3	−2
Heated at the bottom	Model	1	0.050	0.090	0.108	0.124	0.050	0.090	0.108	0.124	0.039	0.072	0.091	0.109
		2	0.099	0.178	0.213	0.245	0.099	0.178	0.213	0.245	0.076	0.141	0.181	0.217
		3	0.147	0.265	0.319	0.367	0.147	0.265	0.319	0.367	0.113	0.210	0.270	0.324
	Experimental	1	0.056	0.087	0.116	0.116	0.047	0.078	0.097	0.116	0.036	0.067	0.082	0.099
		2	0.090	0.164	0.202	0.272	0.087	0.177	0.203	0.226	0.067	0.138	0.171	0.205
		3	0.165	0.265	0.306	0.408	0.136	0.253	0.314	0.388	0.098	0.205	0.278	0.347
	Discrepancies *, %	1	−10	3	−8	7	7	15	10	7	7	7	11	10
		2	10	8	5	−10	14	0	5	9	13	2	6	6
		3	−11	0	4	−10	8	5	1	−6	15	3	−3	−7

* The discrepancies were calculated as the difference between the calculated and measured values, which was referred to the measured value, and given as a percentage.

**Table 2:** Thermal conductivities and densities calculated for different structures.

Structure		Quadrangle and Hexagonal					Triangle				
	Number of	Dimension of closure, mm					Dimension of closure, mm				
	layers	4	5	6	8	10	4	5	6	8	10
		Thermal resistance, m^2^K/W					Thermal resistance, m^2^K/W				
**Heated at the top**	1	0.050	0.070	0.090	0.131	0.171	0.039	0.055	0.072	0.108	0.144
	2	0.099	0.137	0.178	0.260	0.340	0.076	0.107	0.141	0.213	0.287
	5	0.244	0.340	0.441	0.647	0.848	0.186	0.264	0.349	0.529	0.713
	10	0.485	0.677	0.880	1.292	1.693	0.370	0.526	0.696	1.056	1.424
**Heated at the bottom**	1	0.050	0.070	0.090	0.108	0.124	0.039	0.055	0.072	0.091	0.109
	2	0.099	0.137	0.178	0.213	0.245	0.076	0.107	0.141	0.181	0.217
	5	0.244	0.340	0.441	0.529	0.610	0.186	0.264	0.349	0.449	0.538
	10	0.485	0.677	0.880	1.057	1.218	0.370	0.526	0.696	0.895	1.074
		Density, kg/m^3^					Density, kg/m^3^				
**Density**	1	580.5	464.4	387.0	290.3	232.2	725.6	580.5	483.8	362.8	290.3
	2	507.9	406.4	338.6	254.0	203.2	653.1	522.5	435.4	326.5	261.2
	5	464.4	371.5	309.6	232.2	185.8	609.5	487.6	406.4	304.8	243.8
	10	449.9	359.9	299.9	224.9	180.0	595.0	476.0	396.7	297.5	238.0

## Abbreviations

*A*	area of heat transfer, m^2^
*D*	dimension of structure closure, m
*H*	multilayer insulation thickness, m
*K*	thermal conductivity, W/(m·K)
*L*	wall length, m
*R*	thermal resistance, m^2·^K/W
*T*	temperature, K
*q*	heat flux, W/m^2^
**Greek symbols**	
δ	plastic wall thickness, m
ε	emissivity of plastic material
σ	Stefan–Boltzmann constant
**Subscripts**	
a	air
C	cooled side
c	convection
H	heated side
HB	heated at the bottom
HT	heated at the top
i	insulation
L	related to walls that have length L
n	number of layers
p	plastic
r	radiation
w	wall
